# Health risk associated with residential relocation among people who inject drugs in Los Angeles and San Francisco, CA: a cross sectional study

**DOI:** 10.1186/s12889-022-13227-4

**Published:** 2022-04-25

**Authors:** Joey C. Chiang, Ricky N. Bluthenthal, Lynn D. Wenger, Colette L. Auerswald, Benjamin F. Henwood, Alex H. Kral

**Affiliations:** 1 UC Berkeley-UCSF Joint Medical Program, UC Berkeley School of Public Health, 570 University Hall, 94720 Berkeley, CA USA; 2 Department of Preventive Medicine, Keck School of Medicine, University of Southern California, 2001 N. Soto Street, 90032 Los Angeles, CA USA; 3 Behavioral Health Research Division, RTI International, 2150 Shattuck Avenue, Suite 800, 94704 Berkeley, CA USA; 4 Suzanne Dworak-Peck School of Social Work, University of Southern California, Montgomery Ross Fisher Building, 90089-0411 Los Angeles, CA USA

**Keywords:** Residential relocation, Overdose, Violence, Injection drug use, Incarceration

## Abstract

**Background:**

Given the housing instability and frequent residential relocation (both volitional and hegemonic) of people who inject drugs, we sought to determine whether residential relocation (defined as sleeping in a different place in the past 30 days) is associated with health outcomes in a sample of people who inject drugs (PWID).

**Methods:**

We recruited 601 PWID using targeted sampling and interviewed them between 2016 and 2018 in San Francisco and Los Angeles, CA about housing, drug use practices, and service utilization. We then developed multivariable regression models to investigate how residential relocation is associated with violence, health outcomes, and social service access. We analyzed our data between June 2018 and October 2019.

**Results:**

Participants who relocated in the past 30 days had lower odds of being in substance use treatment (Adjusted Odds Ratio [AOR] = 0.62, 95% Confidence Interval [CI] = 0.42, 0.89) and higher odds of nonfatal overdose (AOR = 2.50, CI = 1.28, 4.90), receptive syringe sharing (AOR = 2.26, CI = 1.18, 4.32), severe food insecurity (AOR = 1.69, CI = 1.14, 2.50), having belongings stolen (AOR = 2.14, CI = 1.42, 3.21), experiencing physical assault (AOR = 1.58, CI = 1.03, 2.43), arrest (AOR = 1.64, CI = 1.02, 2.65), and jail (AOR = 1.90, CI = 1.16, 3.13) in the past 6 months when compared to those who did not relocate.

**Conclusions:**

PWID who have relocated in the past 30 days have higher odds of experiencing violence and life- threatening adverse outcomes, and policies that disrupt living circumstances of PWID should be ended in favor of those that support housing stability.

## Introduction/Background

Within North America, people who inject drugs (PWID) experience myriad adverse health outcomes (e.g. infectious diseases, overdoses, drug-related suicidal behaviors, and comorbid psychiatric disorders) when compared to their counterparts who do not inject drugs [1, 2]. In conjunction to these health outcomes associated with injection drug use, PWID are at increased risk of low socioeconomic status, homelessness, and poor access to the medical system, all of which subsequently contribute to increased morbidity and mortality among PWID [3, 4]. Among the multiple mechanisms that link injection drug use to increased morbidity and mortality, housing instability is a risk factor independent of HIV infection or drug use, and may be linked to limited security in more informal living environments [5–7]. Recent studies of PWID in North America found that residential eviction is associated with increased risk of HIV/HCV infection, higher-risk substance use behaviors (e.g. syringe sharing), exposure to violence, as well as all-cause mortality among housing-unstable PWID when compared to their housing-secure counterparts [7–10]. Injection drug use and housing instability are often associated with racial minority identities within the United States, given its lengthy history of discriminatory housing and legislative policies targeting these communities, which has contributed to the disparities in substance use disorders and housing insecurity seen today [11, 12].

The adverse health outcomes associated with housing instability among PWID may be best understood using the concept of ontological security. First described by Laing in the 1960s to characterize traits people with mental health conditions lacked, ontological security was a process where one recognized their “own being as real, alive, whole; as differentiated from the rest of the world in ordinary circumstances so clearly that [their] identity and autonomy are never in question” [13]. Ontological security has been a dynamic concept, as it was redefined by Giddens as a ‘security of being’, a ‘confidence or trust that the natural and social worlds are as they appear to be, including the basic existential parameters of self and social identity’ [14]. Padgett was the first to use ontological security in the field of homelessness, employing it to understand the effects of permanent supportive housing (PSH) on a housing-unstable group of adults. She found recipients were given a space to contemplate “what’s next?” instead of just prioritizing their survival [15]. While ontological security is not solely the process of obtaining a home, as Henwood describes in their study of PSH for unhoused youth: “moving into PSH brought with it Dupuis and Thorns’ four traditional markers of ontological security… to have a place: (1) of social and material constancy; (2) where daily routines can be enacted and carried out; (3) ‘where people feel most in control in their lives because they are free from surveillance;’ and (4) ‘around which identities are constructed’” [16, 17]. While not all people experiencing homelessness or PWID lack ontological security, there were clear benefits associated with permanent supportive housing among previously unhoused youth who received PSH. While ontological security yields health benefits when established, loss of ontological security can also precipitate adverse health effects (e.g. when people are evicted from their primary residence). Adverse health outcomes from ontological insecurity can be seen among housing-unstable populations, but are often more pronounced among specific groups, including people experiencing homelessness and people who inject drugs (PWID) [18].

The growing body of work connecting residential eviction of PWID in North America pairs with policies within major cities of the United States that have resulted in hegemonic relocation of people experiencing homelessness. Within this paper we will use the terms moved and relocated synonymously to describe the volitional or hegemonic movement of study participants. While many aspects of the experience of homelessness warrant volitional residential relocation that may confer mental or physical health benefits (e.g. unsafe or unsanitary living situations, desire to be closer to social supports/food), cities such as San Francisco and Los Angeles, California have been performing encampment sweeps or targeted relocation of people experiencing homelessness (defined by the authors as hegemonic residential relocation) for several years. The Coalition on Homelessness in San Francisco has documented how policies of hegemonic relocation are associated with policing of San Francisco’s unhoused populations [19]. Their survey of 351 respondents found that 46% had their belongings confiscated by city officials, 38% had their belongings destroyed by city officials, and 47% reported that their fear of being searched prevented them from carrying needed belongings [19]. These findings shed insight on how hegemonic residential relocation and policing of street homelessness are inextricably linked and how both negatively impact the ontological security of people who are moved. We are aware of no research that has assessed the association between residential relocation and violence, health, or access to services among PWID that includes people living in informal living situations. This study addresses this gap by investigating how residential relocation may be associated with these outcomes among an unstably housed sample of PWID living in San Francisco and Los Angeles. Both of these cities are particularly suitable for this research given the high number of people who are unstably housed whose primary residence is the street, as well as the high frequency with which city officials move people who are unsheltered [20, 21].

## Methods

### Sampling and recruitment

Survey data from PWID living in San Francisco and Los Angeles were collected for the Change the Cycle (CTC) study, a United States National Institute on Drug Abuse-funded randomized controlled trial performed between 2016 and 2017 to assess the efficacy of a behavioral intervention on injection initiation among PWID. Participants were recruited using targeted sampling of participants from previously identified street, community, and program sites, a method developed by Watters and Biernacki to sample populations who are difficult-to-reach with traditional sampling methods [22–24]. Targeted sampling involves the synthesis of secondary data, such as drug treatment and arrest data, and direct community observation (ethnography) to map out target neighborhood blocks for recruitment. Based on estimates of relevant population size of people who inject drugs, a representative number of individuals are recruited from each area by an outreach worker familiar with the neighborhoods and population. Inclusion criteria for this study required participants to be 18 years of age or older, to self-report injecting drugs in the past 30 days, and to have the evidence of recent drug use confirmed by staff inspection [25]. The interviews were performed at private field sites leased by RTI and USC, which were easily accessible by foot or public transport. After providing written informed consent, survey participants completed a computer-assisted personal interview (Questionnaire Development System, Nova Research, Bethesda, MD) in which trained interviewers spent 40–60 min reading questions aloud and recording the answers on a laptop computer. Participants were remunerated $15 for their time and participation. The six-month follow-up survey for CTC introduced questions concerning housing and relocation, thus it was the dataset for this cross-sectional analysis. The last six-month interview was collected on June 10th, 2018 and the analysis was performed from June 2018 to October 2019. The CTC intervention conducted at baseline included no content that would influence the exposures or outcomes of this analysis. The baseline sample for the CTC study included 979 individuals, of whom 601 participated in the 6-month follow-up survey (316 in San Francisco and 285 in Los Angeles) which included questions about housing status and residential relocation. All protocols were approved by the Institutional Review Board at the University of Southern California.

### Study measures

The main exposure variable was if study participants experienced residential relocation in the last 30 days. We asked each participant “In the last 30 days, how many times did you sleep in a different place or location (same type of place but different location)?” We created both dichotomized (those who moved in the past 30 days compared to those did not move in the past 30 days) and categorical distributions of our relocation variable. The categorical distribution was created by generating approximate quartiles based on frequency of residential relocation, yielding the following move categories: did not relocate, relocated < 3 times, relocated 3–9 times, and relocated > 9 times in past 30 days.

The main outcome variables included exposure to violence, health outcomes, health behaviors, criminal legal system involvement, and access to services. Participants’ exposure to violence was assessed by asking whether they had their belongings stolen (“In the past 6 months, have any of your belongings been stolen?”), experienced physical assault (“In the last 6 months has anybody punched, slapped, kicked, or physically hurt you?”), experienced weaponized assault (“In the past 6 months, has anybody used a knife, gun, club, or other weapon against you?”), or had experienced sexual assault (“In the past 6 months, has somebody used physical force or threats to make you have vaginal sex, anal sex, or oral sex with them?”). The responses to these items were all coded as binary (yes vs. no).

We assessed participants’ health outcomes, health behaviors, and access to services by asking about overdose, injecting with syringes used by others, severe food insecurity, and access to substance use treatment. For overdose, we asked participants “In the last 6 months, have you overdosed?” and coded participant responses as binary (yes vs. no). For injecting with syringes used by others, we asked “In the last 6 months, how many times did you inject using syringes/needles that you know had been used by someone else (including a close friend or lover)?” Responses were coded as binary (whether participants had any injecting with used syringes vs. no injecting with used syringes in the past 6 months). To assess food insecurity during the prior 30 days we used a 10-question scale utilized by Schmitz et al. that consisted of questions about skipping meals, losing weight because of inability to access food, and concern about access to food [26, 27]. Participants were assigned one point for each food insecurity question they endorsed. Participants with 0 to 5 points were designated as not severely food insecure, while participants with 6 to 10 points were designated as severely food insecure. We assessed participants’ access to substance use treatment by asking the following question: “In the last 6 months, have you participated in any type of substance use treatment program (including methadone or alcohol treatment, but excluding NA, AA, or other self-help programs)?” Participant responses were coded as binary (yes vs. no).

Criminal-legal system involvement was assessed by asking participants if they had been arrested (“Have you been arrested in the last 6 months?”), or if they had been incarcerated (“In the last 6 months, have you been held overnight in jail?”). Both outcomes were operationalized as binary (yes vs. no).

We also included several demographic variables as potential confounding factors. These included gender (cismale, cisfemale, transgender, or other), sexual orientation (heterosexual, gay/lesbian, or bisexual), race/ethnicity (White, Latinx, Black, Asian/Pacific Islander, Native American, Mixed Race/Other), homelessness (If respondents considered themselves to be homeless or unstably housed, or not), education (less than high school diploma or high school diploma or more), age (18–29, 30–39, 40–49, or 50 or older), relationship status (single, in a relationship but not living as married, or married/living as married), and monthly income (Less than $1,400, or $1,400 or more).

### Statistical analysis

Descriptive statistics, including means and frequencies, were generated for all study variables. We analyzed the relationship between our relocation variable and our three aggregate outcome variables which were grouped a priori: exposure to violence, health (including health outcomes, health behaviors, and access to services), and involvement with the criminal legal system. Bivariate analyses consisting of odds ratios and *χ*2 tests were conducted to assess associations between the relocation variable and outcome variables. Potential confounding by demographic variables (e.g. self-reported race/ethnicity, gender, income, age) was assessed with two-way *χ*2 tests (p < 0.05) for each outcome variable. Variables found to be associated with both the explanatory variable (residential relocation) and outcome variables were included as potential covariates in multivariable logistic regression models generated for each outcome variable. Covariates not statistically significantly associated with the outcome in the models at *p* < 0.05 were removed in the final models. Data was analyzed in R, version 3.5.3 [28].

## Results

Participants (*N* = 601) were 40% white, 24% Black, 22% Latinx, 7% Asian/Pacific Islander, 1% Native American, and 6% of mixed race; 65% were under the age of 50; 79% earned less than $1401 monthly; 75% identified as homeless or unstably housed; and 19% identified as gay, lesbian, or bisexual (Table 1).

**Table 1:** Demographic and behavioral characteristics of PWID in San Francisco and Los Angeles (*N* = 601)

Characteristics	N (%)
Study Site	
San Francisco	316 (53%)
Los Angeles	285 (47%)
Gender	
Male	440 (73%)
Female	150 (25%)
Transgender	5 (1%)
Other	4 (1%)
Sexual Orientation	
Heterosexual	483 (80%)
Gay, lesbian, or bisexual	116 (20%)
Race/Ethnicity	
White	239 (40%)
Latinx	133 (22%)
Black	142 (24%)
Asian/Pacific Islander	42 (7%)
Native American	6 (1%)
Mixed Race/other	37 (6%)
Education	
Less than high school diploma	170 (28%)
High school diploma or more	429 (72%)
Age	
18–29	93 (15%)
30–39	136 (23%)
40–49	160 (27%)
50 and Older	210 (35%)
Relationship Status	
Single	411 (68%)
In a relationship, but not living as married	92 (15%)
Married or living as married	96 (16%)
Monthly Income	
Less than $1,400	473 (79%)
$1,400 or More	128 (21%)
Experiencing homeless currently	451 (75%)
Relocated in the past 30 days	427 (72%)
Relocation by Approximate Quartile	
Did not relocate in past 30 days	167 (28%)
Relocated 1–2 times in past 30 days	117 (20%)
Relocated 3–9 times in past 30 days	159 (27%)
Relocated more than 9 times in past 30 days	147 (25%)
Experience of violence in the past 6 months	
Having belongings stolen	423 (70%)
Experiencing physical assault	210 (35%)
Experiencing weaponized assault	115 (19%)
Experiencing sexual assault	23 (4%)
Health outcomes, related behaviors, and access to treatment in the past 6 months	
Overdose	85 (14%)
Severe food insecurity	238 (39%)
Receptive syringe sharing	81 (13%)
Substance use treatment	196 (33%)
Criminal Legal System Involvement in the past 6 months	
Arrest	169 (28%)
Jail	162 (27%)

In this sample, 72% reported residential relocation at least once in the past 30 days. The proportion of relocation by counts was as follows: 28% had not relocated, 20% relocated 1–2 times, 27% relocated 3–9 times, and 25% relocated over 9 times. Participants’ experience of violence in the prior 6 months was extensive: 70% of participants had their belongings stolen, 35% were subjected to physical assault, 19% were subjected to weaponized assault, and 4% were subjected to sexual assault. Examining the frequency of health outcomes, health related behaviors, and access to treatment, 17% of participants had overdosed in the past 6 months, 13% of participants reported receptive syringe sharing in the past 6 months, 39% of participants were experiencing severe food insecurity in the past 30 days, and 33% of participants received substance use treatment in the past 6 months. For criminal legal system outcomes, 28% of participants reported arrest and 27% of all participants had been held in jail overnight in the prior 6 months.

Bivariate analyses (summarized in Tables 2, 3, and 4) revealed that participants who had to relocate in the past 30 days had higher prevalence of reporting stolen belongings, physical assault, and weaponized assault when compared to participants who had not been moved. Participants who moved in the past 30 days also had higher prevalence of receptive syringe sharing, lower prevalence of accessing substance use treatment, and higher prevalence of arrest in the past 6 months when compared to participants who had not been moved. Furthermore, participants’ reports of violence, negative health outcomes (overdose, receptive syringe sharing, severe food insecurity, and decreased substance use treatment), and of criminal legal system involvement increased with the frequency of residential moves.

**Table 2:** Residential relocation and frequency of violence outcomes among PWID in Los Angeles and San Francisco (*N* = 594)^a^

	Residential Relocation Status	Having Belongings Stolen	Experiencing Physical Assault	Experiencing Weaponized Assault	Experiencing Sexual Assault
Bivariate Analysis	Relocated in past 30 days (*n* = 427)	327 (77%) ***	167 (40%) **	93 (23%) *	18 (78%)
	Did not relocate (*n* = 167)	91 (56%)	255 (23%)	21 (13%)	403 (71%)
Categorical Analysis	Relocated > 9x (*n* = 121)	127 (87%) ***	76 (52%) ***	49 (34%) ***	8 (6%)
	Relocated 3-9x (*n* = 189)	129 (83%)	64 (41%)	30 (19%)	7 (4%)
	Relocated < 3x (*n* = 117)	68 (59%)	26 (22%)	13 (11%)	3 (3%)
	Did not relocate (*n* = 167)	91 (54%)	39 (23%)	21 (13%)	5 (3%)

*= *χ*2 test between residential relocation and outcome significant at *p* < 0.05

**= *χ*2 test between residential relocation and outcome significant at *p* < 0.01

***= *χ*2 test between residential relocation and outcome significant at *p* < 0.001

^a^ fewer than 601 individuals responded to this question

**Table 3:** Residential relocation and frequency of health outcomes among PWID in Los Angeles and San Francisco (*N* = 594) ^a^

	Residential Relocation Status	Overdose	Receptive Syringe Sharing	Severely Food Insecure	Drug Treatment
Bivariate Analysis	Relocated in past 30 days (*n* = 427)	73 (17%) **	67 (16%) **	184 (47%) *	126 (30%) *
	Did not relocate (*n* = 167)	11 (7%)	12 (7%)	51 (34%)	68 (41%)
Categorical Analysis	Relocated > 9x (*n* = 121)	30 (21%) **	28 (21%) **	84 (60%) ***	35 (24%) *
	Relocated 3-9x (*n* = 189)	24 (15%)	26 (17%)	61 (43%)	48 (31%)
	Relocated < 3x (*n* = 117)	19 (16%)	13 (11%)	36 (33%)	43 (37%)
	Did not relocate (*n* = 166)	11 (7%)	12 (7%)	51 (31%)	68 (41%)

*= *χ*2 test between residential relocation and outcome significant at *p* < 0.05

**= *χ*2 test between residential relocation and outcome significant at *p* < 0.01

***= *χ*2 test between residential relocation and outcome significant at *p* < 0.001

^a^ fewer than 601 individuals responded to this question

**Table 4:** Residential relocation and frequency of criminal-legal outcomes among PWID in Los Angeles and San Francisco (*N* = 594) ^a^

	Residential Relocation Status	Arrest	Jail
Bivariate Analysis	Relocated in past 30 days (*n* = 427)	138 (32%) ***	136 (32%) ***
	Did not relocate (*n* = 167)	28 (17%)	25 (15%)
Categorical Analysis	Relocated >9x in the past 30 days (*n* = 119)	61 (42%) ***	56 (39%) ***
	Relocated 3-9x in the past 30 days (*n* = 186)	51 (33%)	53 (34%)
	Relocated <3x in the past 30 days (*n* = 115)	25 (22%)	26 (23%)
	Did not relocate (*n* = 156)	28 (17%)	25 (15%)

* *χ*2 test between residential relocation and outcome significant at *p* < 0.05

** *χ*2 test between residential relocation and outcome significant at *p* < 0.01

*** *χ*2 test between residential relocation and outcome significant at *p* < 0.001

^a^ fewer than 601 individuals responded to this question

We created separate multivariable logistic regression models for each outcome variable (Table 5). As compared to individuals who had not moved, we found that the relationships between residential relocation and outcome variables were statistically significant for all outcomes except for weaponized violence. Participants who relocated in the past 6 months had higher odds of overdose (Adjusted Odds Ratio [AOR] = 2.50, 95% Confidence Interval [CI] = 1.28, 4.90), receptive syringe sharing (AOR = 2.26, 95% CI = 1.18, 4.32), severe food insecurity in the last 30 days (AOR = 1.69, 95% CI = 1.14, 2.50), having belongings stolen (AOR = 2.14, 95% CI = 1.42, 3.21), experiencing physical assault (AOR = 1.58, CI = 1.03, 2.43), arrest (AOR = 1.64, CI = 1.02, 2.65), and jail (AOR = 1.90, 95% CI = 1.16, 3.13), and lower odds of substance use treatment (AOR = 0.62, 95% CI = 0.42, 0.89) in the past 6 months when compared to those who did not relocate.

**Table 5:** Multivariable analysis of residential relocation and outcomes among PWID in San Francisco and Los Angeles (*N* = 590)^d^

Outcome	Any relocation in the past 30 days Adjusted Odds Ratio (95% Confidence Interval)
Overdose	2.50 (1.28, 4.90) ** ^a,b^
Receptive syringe sharing	2.26 (1.18, 4.32) * ^c^
Severe food insecurity	1.69 (1.14, 2.50) **
Substance use treatment	0.62 (0.42, 0.89) *
Having belongings stolen	2.14 (1.42, 3.21) *** ^a,b,c^
Experiencing physical assault	1.58 (1.03, 2.43) * ^a^
Experiencing weaponized assault	1.56 (0.91, 2.67) ^a,b^
Arrest	1.64 (1.02, 2.65) * ^a^
Jail	1.90 (1.16, 3.13) * ^a^

*=Significant at *p* < 0.05

**=Significant at *p* < 0.01

***=Significant at *p* < 0.001

^a^ Controlled for age

^b^ Controlled for White race

^c^ Controlled for self-identification as gay, lesbian, or bisexual

^d^ fewer than 601 individuals responded to this question

## Discussion

To our knowledge, this is the first study to demonstrate associations between residential relocation and negative health and social outcomes among a sample of unstably housed PWID, of which a majority were experiencing homelessness. We found that any residential movement in the past 30 days was directly associated with increased odds of experiencing violence, life-threatening health outcomes, high-risk health behaviors, decreased access to services, and criminal-legal system involvement.

People in our study who relocated in the prior 30 days had higher odds of experiencing violence within the past 6 months. This phenomenon corroborates work done by the San Francisco Coalition on Homelessness, which found that individuals experiencing homelessness and living on the street were likely to have their belongings searched, destroyed, or confiscated by city officials [19]. A significant association may not have been detected between displacement and weaponized violence due to the increased lethal potential for weaponized attacks, resulting in a differential loss to follow-up, as people who were severely wounded or died by weaponized violence cannot have participated in the study. Our research supports these claims and finds that individuals who relocated in the prior 30 days also had greater odds of experiencing physical assault in the past 6 months. These greater odds may be explained either by individuals leaving violent situations or the effect of relocation on ontological security, as they may be forced to relocate to unsafe or unfamiliar surroundings with predators or discriminatory policing. Future research should be centered around whether a causal relationship between residential relocation and violence exists and further investigate if the associations seen in this analysis are specifically associated with hegemonic operations such as encampment sweeps. Such work should engage the voices of people experiencing homelessness to clarify the mechanisms by which residential movement leads to increased harm.

We also found that residential relocation in the past 30 days was associated with potentially life-threatening health outcomes such as overdose, negative health related behaviors such as receptive syringe sharing, and low treatment utilization. When PWID experience frequent relocation, they may be reticent to keep more syringes on their person for fear of persecution by law enforcement [19, 29, 30]. Furthermore, if people are move farther distances, logistical barriers (e.g. increased distance accessing their usual syringe services program) may interfere with receiving sterile syringes. Our findings draw parallels to work done by Pilarinos et al. that found that recent residential eviction was associated with syringe sharing among street-involved youth in Vancouver, Canada, as well as work done in Kabul, Afghanistan by Todd et al. that found syringe sharing and other risk behaviors were markedly increased during periods of conflict and social unrest [10, 31]. The process of residential movement may be similarly prohibitive for people who utilize medication assisted treatment (MAT). As methadone was the most common treatment option in San Francisco and Los Angeles and required daily dosing, residential relocation may have created physical or logistical barriers to making methadone appointments. Relocation may also worsen overdose outcomes by establishing barriers to naloxone access by confiscation of belongings or by disruption of communities able to administer naloxone in the event of an overdose [32]. Further research regarding the effect of residential relocation of persons experiencing homelessness and PWID on their health-related behaviors, health outcomes and access to services should delineate the mechanism between residential relocation and poor health outcomes. Since we found age, self-identification as White or Latinx, and self-identification as gay, lesbian, or bisexual to be confounders in the relationship between residential relocation and several outcomes, subsequent work should elucidate the effects of race, gender, and sexuality on relocation.

Recent residential relocation was also associated with increased odds of criminal-legal system involvement, as individuals who moved in the past 30 days had higher odds for both arrest and incarceration. This association with the criminal-legal system is likely due to police involvement in the movement of people who experience homelessness. Furthermore, when people relocate, they may also lose their belongings due to a disruption of their residential organization. This in turn could drive them to engage in criminal behaviors to meet their basic needs. The findings from our work concur with existing literature on criminalization of homelessness that finds criminalization costly to its targets and to taxpayers [33, 34]. Further research assessing the relationship between residential relocation and criminal legal system involvement should be directed toward uplifting the voices of people experiencing homelessness to understand the harm of responding to homelessness with punitive policies, or studying the effect of new permanent supportive housing on the rates of relocation and police engagement.

The strong associations between residential relocation and experience of violence do not suggest temporality between the two variables. While some study participants may have moved following an experience of violence to escape from its source, participants may have also moved to a situation that was deemed not safe for them. Further research must be done in this realm to identify the temporal relationship between residential relocation and violence. While temporality cannot be assessed, these findings suggest that solutions should urgently be focused on shifting funds away from punitive approaches (e.g. police and Department of Public Works sweeps) and instead should be focused on investing in permanent housing, supportive services, and linkages to care (e.g. low-barrier extended-stay shelters, known locally as navigation centers, social work first-responders, and case managers). Solutions should also be centered around restoring ontological security to those who experience hegemonic residential relocation. This includes utilizing a housing-first approach, reducing unnecessary movement of individuals experiencing homelessness, investing in additional syringe service program and MAT sites, and developing supervised injection sites, which have been found to lower costs and effectively prevent syringe sharing, overdoses and the spread of infections [35–38].

## Limitations

The results of this study must be considered along with potential methodological limitations. We employed cross-sectional analyses, which were not able to assess the temporality between explanatory and outcome variables. These cross-sectional data was pulled from the 6-month follow-up of the larger study and could have incurred selection bias for individuals able to attend follow-up visits. Given the mismatch in duration between the dependent variable (relocation) and the outcomes of interest, it is possible certain outcomes (e.g. violence, health consequences) influenced the participants’ decision to move. If the intervention in the CTC study was pertinent to the themes of this analysis, this may have also influenced the outcomes. Fortunately, this is unlikely given the CTC intervention was not focused on housing stability and the intervention itself was randomized. It is essential to reiterate that our exposure variable likely captured moves not due to hegemonic operations (e.g. encampment sweeps or targeted movement of unhoused individuals), but rather due to individual autonomy or self-agency (e.g. leaving an unsafe living situation). While our findings suggest that residential instability among PWID is associated with these adverse outcomes, further work needs to be done to understand the health effects of policing unhoused communities. Additionally, outcome variables were assessed over the prior 6 months instead of the 30-day-period used for the explanatory variable. We believe that study participants may have experienced some of these outcomes prior to an episode of a residential move, further research must be done to clarify the temporal relationship between these variables. The study was also dependent upon self-reported measures. Self-reporting could have introduced desirability bias, which may have caused under-reporting.

Our findings suggest that the residential relocation of PWID may be disruptive and carries potential for harmful and life-threatening consequences for PWID. While further research must be done to clarify causal relations between residential relocation and health outcomes, policies that disrupt living circumstances of PWID should be ended in favor of those that support housing stability.

## Data Availability

The datasets used and/or analysed during the current study are available from the corresponding author on reasonable request.

## Abbreviations

CTC: Change the Cycle Study
MAT: Medication Assisted Treatment
PWID: People Who Inject Drugs
PSH: Permanent Supportive Housing
